# First case report of diagnosis of extrapancreatic solid pseudopapillary tumor with SMA invasion in a 47-year-old man: a case report and literature review

**DOI:** 10.3389/fsurg.2024.1409564

**Published:** 2024-08-06

**Authors:** Aijing Zhang, Kaibin Wang, Xiaohan Tian, Shuhai Chen, Jianwei Xu, Han Liu, Lei Wang, Feng Li

**Affiliations:** ^1^ Department of Urology, Qi Lu Hospital of Shandong University, Jinan, China; ^2^ Department of Orthopedics, Qi Lu Hospital of Shandong University, Jinan, China; ^3^ Department of Breast Surgery, General Surgery, Qi Lu Hospital of Shandong University, Jinan, China; ^4^ Department of Pancreatic Surgery, General Surgery, Qi Lu Hospital of Shandong University, Jinan, China; ^5^ Department of Gastroentero-Pancreatic Surgery, Qilu Hospital (Qingdao), Cheeloo College of Medicine, Shandong University, Qingdao, Shandong, China

**Keywords:** solid pseudopapillary tumor of the pancreas (SPT), pancreatic cancer, extrapancreatic tumor, ectopic SPT, vascular invasion

## Abstract

**Background:**

Solid pseudopapillary tumor of the pancreas (SPT) is a rare low-grade malignant tumor predominantly observed in young women without significant clinical symptoms. While most SPTs occur in the pancreatic region, rare cases have occurred in the retroperitoneum, making the diagnosis of ectopic SPTs difficult.

**Case presentation:**

Herein, we report a rare case of an extrapancreatic solid SPT with superior mesenteric artery (SMA) involvement in a 47-year-old man together with a literature review to provide context with clinical information, CT and a literature review.

**Conclusions:**

This case may provide a practical approach for the diagnosis of ectopic SPT, especially for patients with vascular invasion.

## 1 Introduction

Among all pancreatic tumors, solid pseudopapillary tumor of the pancreas (SPT) is rare and has low malignant potential, accounting for approximately 0.9%–2.7% of pancreatic tumors in adults (1). As described by Frantz in 1959, SPT mainly occurs in young women (mean age 28 years) and its frequency has recently increased (2). SPT is usually recognized as a large mass with a cystic component predominantly located in the tail of the pancreas (3, 4). Patients with SPTs typically have presented nonspecific symptoms such as abdominal discomfort, nausea, vomiting, weakness, or pain (5). The recommended therapy for SPTs is surgical resection through open or laparoscopic resection, depending on tumor location and/or size (6, 7). The overall survival following surgery is generally favorable with low recurrence for patients with SPTs (8). However, SPTs outside the pancreas are exceedingly rare and this is the first case to report a histologically documented case of a male patient with extrapancreatic SPT with superior mesenteric artery (SMA) invasion. Given the unusual combination of extrapancreatic location, male gender and solid nature without a cystic component, it is instructive to document this case to highlight the diagnosis and treatment of SPTs.

## 2 Case presentation

**Figure 1:**
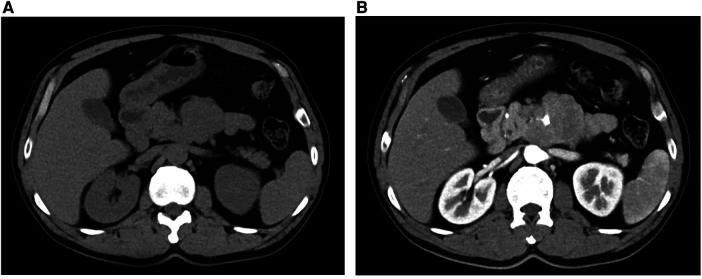
CT (**A**) and contrast-enhanced CT (**B**) showing an occupying lesion below the neck of the pancreas.

A 47-year-old man with cerebral infarction 1 year prior with a 2-day history of intermittent abdominal pain, associated with anorexia. A thoracic CT scan revealed an occupying lesion below the neck of the pancreas (Figure 1A), which on contrast-enhanced CT was seen to be a 7.7 × 6.0 cm lesion in the vicinity of the left aspect of the pancreatic uncinate process, encasing the SMA and some of its branches (Figure 1B). This raised suspicion for a pancreatic uncinate of retroperitoneal to neoplasm. The radiology department advised that the lesion be biopsied. Blood parameters, including CEA, CA-199, AFP and PSA were normal (Table 1), which were consistent with previous cases of SPTs even with metastases (9), though IL-6 was raised at 9.06 pg/L in this case. A primary diagnosis of a pancreatic space-occupying lesion was made at the very beginning of the study due to the CT findings, despite the presence of normal tumor markers. Following detailed discussion between the physicians and patient, a laparoscopic retroperitoneal tumor biopsy was suggested to determine whether the lesion was malignant or benign because the SMA was surrounded by this lesion, limiting the opportunity for complete resection of the tumor.

**Table 1:** Patient's laboratory results.

	On admission	Reference range	Unit
RBC	5.34	4.3–5.8	10^9^/L
HGB	158	130–175	g/L
MCV	93.5	82–100	Fl
Platelets	246	125–350	10^9^/L
CRP	<0.20	0–10	mg/L
LDH	226	120–230	U/L
D-dimer	0.32	<0.50	μg/ml
Creatine	103	62–115	μmol/L
Urea	6.71	2.3–7.8	mmol/L
APTT	31.9	26–42	S
Fibrinogen	5.34	2–4	g/L
TBIL	13.9	5–21	U/L
ALT	40	9–50	U/L
AST	33	15–40	U/L
CA19-9	13.66	0–37	U/ml
NSE	23.91	0–25	ng/ml
CEA	2.93	0–5	ng/ml
CA242	6.02	0–20	U/ml
AFP	5.52	0–7	ng/ml
Free-PSA	0.11	0–1	ng/ml
TPSA	0.37	0–4	ng/ml
CA125	4.36	0–35	U/ml
CA15-3	4.74	0–28	U/ml
CYFRA21-1	1.53	0–5	ng/ml
SCCA	0.41	0–1.5	ng/ml
CA-724	1.16	0.00–6.90	U/ml

RBC, red blood cells; HGB, haemoglobin; MCV, mean corpuscular volume; CRP, C-reactive protein; LDH, lactate dehydrogenase; APTT, activated partial thromboplastin time; TBIL, total bilirubine; ALT, alanine aminotransferase; AST, aspartate aminotransferase; NSE, neuron specific enolase; CEA, carcinoembryonic antigen; AFP, alpha-fetoprotein gene; Free-PSA, free-prostate-specific antigen; TPSA, total prostate-specific antigen; SCCA, squamous cell carcinoma antigen.

**Figure 2:**
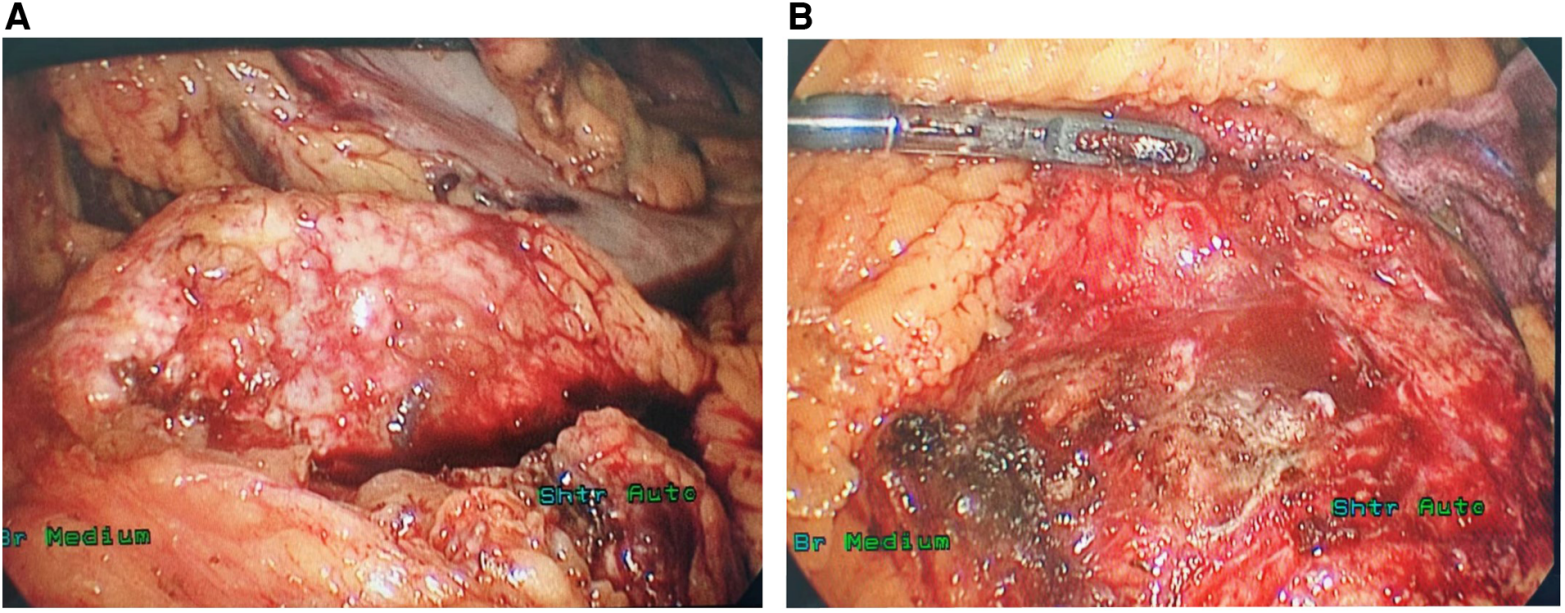
The ectopic SPT surrounded the SMA outside the pancreas (**A**) and no lesion was identified in the pancreas during the operation (**B**).

During the surgery, ascites was not found in the abdominal cavity. No abnormal nodules were observed on the surface of the liver, abdominal wall, pelvis, greater omentum, intestinal walls or mesentery, suggesting no metastasis. After opening the gastrocolic ligament, no obvious space-occupying lesion was observed on the surface of the pancreas. Via the approach of the inferior colon, a large, solid, firm, fixed and highly vascularized tumor was observed behind the root of the mesentery, completely encasing the mesentery (Figures 2A,B). After opening the retroperitoneum, the tumor was carefully biopsied to avoid damage to the SMA. The tumor specimen was then sent for rapid pathological examination, which considered neuroendocrine tumor.

**Figure 3:**
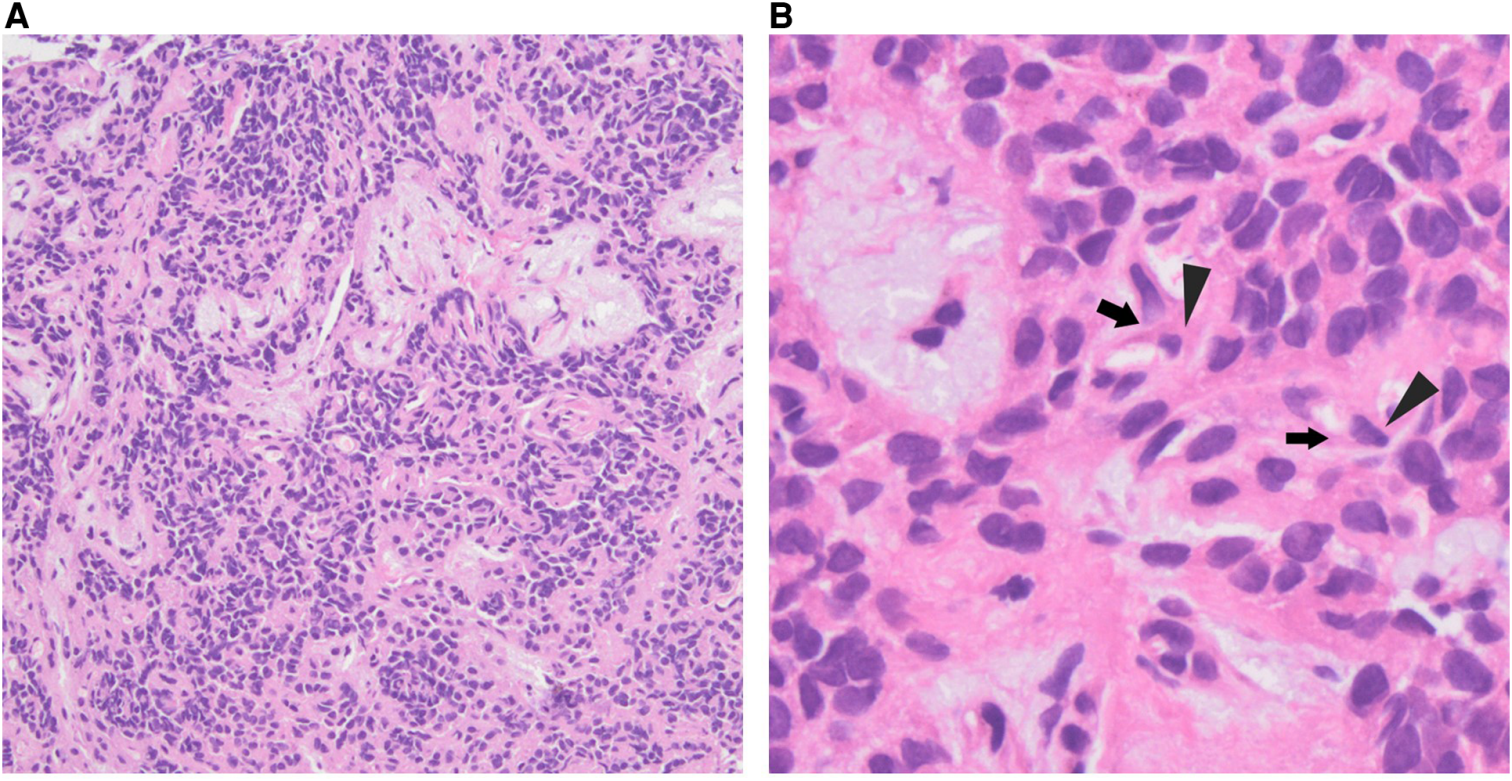
Histological examination showing characteristics of SPT: papillary structures (**A**) and cercariform cells (**B**): slight nuclear atypia (triangles) and delicate elongation of the cytoplasm (arrows). (hematoxylin-eosin, original magnifications ×200[a], ×400[b]).

The standard histopathological report revealed that the neoplasm corresponded to the SPT. The tumor specimen measured approximately about 0.8 × 0.6 × 0.3 cm grossly and subsequent histopathology revealed cercariform cells with pseudopapillary formations (Figures 3A,B). The tumor cells expressed PR(+),β-Catenin(+), LEF1(+), CD10(+), CD56(+), P53(+, 5%), Syn(+), ATRX(+), DAXX(+), MGMT(+), Rb(+), CgA(−), SSTR2(0), CK(−), INSM1(−), CD20(−), CD79a(−) and Ki-67 positive rate 5%.

During the follow-up of 3 months after surgery, the patient experienced a favorable recovery revealing no signs of further progression. This patient only takes Creon regularly, with a satisfactory management of blood glucose levels.

## 3 Discussion

SPT is regarded as a rare tumor, accounting for approximately 0.9%−2.7% of pancreatic tumors. Grossly, SPT is characterized by an encapsulated mass with internal hemorrhage, cystic degeneration and calcification (10). Various names have been used to describe this rare neoplasm such as Frantz tumor and solid and papillary epithelial neoplasm. Moreover, the tumor has a predilection for females in a ratio of 10:1. Fewer than 30 cases of extrapancreatic SPTs have been described. Most of the ectopic SPTs are located in the mesocolon (11), while others have been reported in ovary (12), and testis (13). To the best of our knowledge, this is the first report of ectopic SPT that was located in the retroperitoneum which at the same time showed SMA invasion.

In this case, a male patient diagnosed with extrapancreatic SPT was reported (10). Radiological investigations demonstrated a 7.7 × 6.0 cm tumor, encasing the SMA and its branches. Pathological examination further confirmed the diagnosis of ectopic SPT. We conclude that to diagnose ectopic SPT, histopathological examination is regarded as the gold standard, although most SPTs are indicated by hypodense cystic lesions on CT and normal tumor markers. To be specific, contrast-enhanced CT usually reveals an encapsulated large mass with a thick wall or cystic components inside (11).

Currently, laparoscopic distal pancreatectomy is a widely used approach for SPT resection, particularly for young women. Other therapies, such as gemcitabine treatment, radiotherapy and hormonal therapy are also used for unresectable SPTs, with no consensus on their treating values temporally (14, 15). Due to the rarity of reported ectopic SPTs, although complete surgical excision is curative in SPT patients limited to the pancreas, more detailed guidelines for ectopic SPTs are still lacking. For this patient, the SPT was derived from the retroperitoneum encapsulating the root of the SMA, indicating difficulty in subsequent resection. Thus, the laparoscopic retroperitoneal tumor biopsy approach can be a better alternative. In addition, the treatment decision before surgery is quite difficult due to the complexity of the pancreatic tumor. The primary treatment of cytoreduetive surgery combined with chemotherapy and immunotherapy may be considered if the lesion is aggressive. Considering the promising long-term prognosis of SPT patients, even those with metastasis, for whom a 10-year disease-specific survival rate of 96% has been reported (15), additional treatments for this patient may include chemotherapy, hormone therapy, radiofrequency ablation and cryoablation, although the benefit of these treatments is poorly understood. Furthermore, regular clinical ultrasonographic investigations of this patient are also recommended for monitoring this tumor progression. We still recommend a two-stage cytoreductive surgery of SPT for this patient if he is agreeable to it.

Based on our adequate experience of laparoscopic techniques, we successfully biopsied this tumor which at pathology work-up was shown to be an SPT. A high Ki-67 positive rate may predict the malignant potential and poor prognosis of SPT (2), and we expect this patient with a 5% Ki-67 positive rate to have a long survival.

## 4 Conclusion

SPT is a rare tumor that occurs mainly in young women. Exploratory laparotomy/laparoscopy with biopsy and frozen section is recommended for such tumors in order to inform next steps.

## Data Availability

The original contributions presented in the study are included in the article/Supplementary Material, further inquiries can be directed to the corresponding author.
